# Antioxidant and Anti-Inflammatory Activities of *Latilactobacillus curvatus* and *L. sakei* Isolated from Green Tripe

**DOI:** 10.3390/nu17152464

**Published:** 2025-07-28

**Authors:** Ga Hun Lee, Sung Hyun Choi, Yong Hyun Lee, Jae Kweon Park

**Affiliations:** Department of Life Sciences, College of Bionano, Gachon University, Seongnamdaero 1342, Seongnam-si 461-701, Republic of Korea

**Keywords:** antioxidant, anti-inflammatory, chitosan, green tripe, *Latilactobacillus*

## Abstract

Background/Objectives: Green tripe (GRET) is rich in essential fatty acids, vitamins, calcium, phosphorus, and other nutrients and contains various beneficial microorganisms, including lactobacillus, along with feed components consumed by ruminants. Methods: In this study, *Latilactobacillus sakei* and *L. curvatus* were isolated from GRET and evaluated for their potential as probiotics, focusing on their anti-inflammatory properties and ability to modulate inflammatory responses. Results: When heat-killed *L. sakei* or *L. curvatus* (10^8^ CFU/mL) and their metabolites (0.5 mg/mL) were applied to RAW 264.7 macrophages stimulated with LPS, nitric oxide (NO) production was reduced by approximately 10–35% and 2–11%, respectively. Furthermore, the expression levels of key anti-inflammatory cytokines, TNF-α and IL-6, were suppressed by more than 5%. These effects were not due to cytotoxicity but instead due to genuine anti-inflammatory activity. In addition, both strains exhibited antioxidant activity, as demonstrated by their performance in ABTS and FRAP assays. Conclusions: These findings suggest that *L. sakei* and *L. curvatus* have significant antioxidant and anti-inflammatory properties, highlighting their potential as probiotics and prebiotics. Moreover, these newly isolated strains from GRET are expected to serve as valuable functional ingredients for developing health-promoting foods and dietary supplements.

## 1. Introduction

Probiotics are defined as live microorganisms that, when administered in adequate amounts, confer health benefits to the host. They are primarily consumed in the form of live microbial dietary supplements. Most probiotics belong to the group of *Lactobacillus* and are known to provide a wide range of health-promoting effects [1,2,3]. The beneficial effects of probiotics include improving gut health, alleviating symptoms associated with lactose intolerance, and reducing the risk of certain diseases [4,5,6]. These effects are mediated through several mechanisms, including modulation of the immune system, competitive exclusion of pathogenic bacteria, and prevention of pathogen adhesion and colonization [7,8,9]. For probiotics to exert their beneficial effects, specific physiological characteristics are required. These include resistance to acidic and bile environments, the ability to adhere to mucosal epithelial surfaces, antimicrobial activity against pathogenic bacteria, and bile salt hydrolase activity.

Common bacterial genera employed as probiotics include *Lactobacillus*, *Bifidobacterium*, *Escherichia*, *Enterococcus*, *Bacillus*, and *Streptococcus*, etc. [10,11,12]. Within the *Lactobacillus* genus, strains such as *L. rhamnosus GG*, *L. sakei*, and *L. curvatus* are widely utilized. *L. rhamnosus GG* (LGG), first patented in 1989, is one of the most extensively studied probiotic strains [13]. LGG is known to possess both antioxidant and anti-inflammatory activities [14,15,16]. In addition, LGG forms a protective biofilm on the intestinal mucosa, enhances the survival of intestinal villi, reduces apoptosis of intestinal epithelial cells, and preserves cytoskeletal integrity, thereby contributing to improved gut health [14,17,18].

Green tripe (GRET) refers to the first stomach (rumen) of ruminant animals, where ingested feed undergoes partial digestion with the assistance of resident bacteria. GRET is composed of fat, protein, calcium, and other nutrients, and contains forage such as grass consumed by the animals, providing a rich source of essential fatty acids, vitamins, calcium, phosphorus, and other micronutrients [19,20]. GRET harbors a diverse microbial ecosystem, including bacteria, archaea, viruses, and fungi. Among the bacterial communities, *Lactobacillus* is prominently present [21,22,23]. *Lactobacillus* strains identified in GRET include *L. casei*, *L. rhamnosus*, *L. fermentum*, and *L. johnsonii*. These strains exhibit resistance to antibiotics, high tolerance to bile salts and low-pH environments, and the ability to adhere to intestinal mucosal surfaces, thereby contributing to the maintenance of a balanced gut microbiota [24,25,26]. In addition, lactic acid-producing bacteria isolated from green tripe have been shown to exhibit notable antimicrobial activity. Cell-free supernatants of *E. avium*, *S. lutetiensis*, and *S. equinus* strains isolated from GRET demonstrated inhibitory effects against pathogenic microorganisms such as *E. coli* and *S. aureus* [27]. These strains also exhibited tolerance to bile salts and low pH, further supporting their probiotic potential. Many *Lactobacillus* and lactic acid-producing bacteria isolated from GRET have the potential to promote gut health and inhibit pathogenic microorganisms [23,28]. As such, GRET is increasingly being recognized not only as a nutritional source but also as a promising material for probiotic applications.

*L. sakei* is a Gram-positive, anaerobic bacterium commonly isolated from a variety of plant-based and animal-based fermented foods and is predominantly found in meat products. Metabolites produced by *L. sakei* have been reported to effectively inhibit the growth of pathogenic bacteria and exhibit notable antioxidant and anti-inflammatory activities [29,30,31]. Due to these physiological properties, *L. sakei* is widely utilized as a probiotic and is also employed as a beneficial microorganism in companion animal feed supplements. *L. sakei* can survive and thrive in extreme environmental conditions, such as low temperature, low pH, high salinity, low water activity, and radiation. Moreover, it has been demonstrated to produce bioactive metabolites by utilizing the nutrients available in meat [32,33]. *L. curvatus* is a Gram-positive lactic acid bacterium isolated from various sources, including sauerkraut, sourdough, fish, and meat. It is known to produce bacteriocins and is highly adaptable to meat environments, where it contributes to meat maturation and the development of desirable flavor profiles [34,35]. *L. curvatus* has been reported to exhibit anti-inflammatory activity by suppressing pro-inflammatory cytokines, and it also demonstrates antioxidant and immunostimulatory effects. Furthermore, *L. curvatus* has shown antimicrobial and anti-biofilm activities against pathogenic bacteria such as *Streptococcus mutans*, indicating its efficacy in inhibiting harmful microorganisms [36,37,38].

There is little research on the microbial diversity and efficient utilization of GRET. Therefore, we aimed to identify microbial species from GRET that could otherwise be simply discarded and evaluate their utility as biomass. In this study, twelve strains of *Lactobacillus* species were isolated from GRET, and *Latilactobacillus curvatus* (Lc) and *L. sakei* (Ls) were selected for further investigation. The objective of this study was to evaluate whether the heat-killed cells and metabolites of these strains exhibit antioxidant and anti-inflammatory activities, thereby validating their potential efficacy as probiotics. Assessing the antioxidant and anti-inflammatory activities of the heat-killed cells and their metabolites provides essential foundational data for understanding the probiotic potential of these strains. Therefore, this study aims to analyze the bioactive components of Lc and Ls and to determine their potential application as probiotics.

## 2. Materials and Methods

### 2.1. Chemicals

Acetic anhydride, 2,2′-azino-bis (3-ethylbenzothiazoline-6-sulfonic acid) di-ammonium salt (ABTS), 1,1-diphenyl-2-picrylhydrazyl (DPPH), 4-hydroxy benzyl hydrazide (PAHBAH), and 2,4,6-tri(2-pyridyl)-s-triazine (TPTZ) were purchased from Sigma Chemical Co. (St. Louis, MO, USA). A molecular porous membrane tube (MWCO 12~14 kDa) for material dialysis was purchased from Spectrum Lab, Inc. (Rancho Dominguez, CA, USA). For quantitative analysis, high-purity glucose, D-glucosamine (GlcN), and *N*-acetyl-D-glucosamine (GlcNAc) were purchased from Sigma Chemical Co. (St. Louis, MO, USA). All other essential reagents used in this study were of the highest purity possible.

### 2.2. Biochemical Characterization: Determination of Reducing Sugar, Total Carbohydrate, and Protein Content

#### 2.2.1. Preparation of Active Molecular Chitosan

Active molecular chitosan (AMC, molecular weight 1.0–3.0 kDa) was prepared by enzymatic treatment of high molecular weight chitosan (HMWC, 98% DD, approximately 2000 kDa; Sokcho Trading Co., Ltd., Sokcho, Republic of Korea) according to a conventional method [39]. Briefly, 2 g of high molecular weight chitosan (HMWC) was dissolved in 100 mL of 2% (*v*/*v*) acetic acid at room temperature for 24 h. The chitosan solution was dialyzed against 2 L of distilled water to remove residual acetic acid. Then, it was dialyzed a second time against 1 L of 25 mM sodium acetate buffer (pH 4.2), and 5 U of chitosanase from *Streptomyces griseus* purchased from Sigma Chemical Co. (St. Louis, MO, USA) was added and reacted at 37 °C for 72 h. The molecular weight range was also performed as described in a previous paper.

#### 2.2.2. Quantification of Reducing Sugar Content

The previous method was slightly modified to determine the reducing sugar content in the sample [40]. Briefly, 16 mM PHABAH (4-Hydroxy-benzhydrazide) solution in ethanol and the samples obtained at each time point were mixed in a consistent ratio, and the mixture was heated at 100 °C for 5 min. After naturally cooling at room temperature for 5 min, the reaction solution was centrifuged at 13,000 rpm for 5 min to remove insoluble substances. The absorbance was measured at 405 nm using a UV spectrometer (InfiniteM200 Pro Nano-quant, TECAN, Untersbergstrasse, Austria). The amount of reducing sugar was quantified using glucose as a standard in the range of 0–10 mM.

#### 2.2.3. Quantitative Analysis of Total Carbohydrates and Protein

The total carbohydrate content in the lactic acid bacteria culture medium and heat-treated bacterial cells was measured using the sulfuric acid–phenol quantitative method [41]. The protein content of the culture supernatant and cells disrupted by heat treatment was measured using bovine serum albumin (BSA) according to the conventional Bradford method.

### 2.3. Isolation of GRET-Derived Beneficial Bacteria and Separation of Intracellular Metabolites Through Heat Treatment

The following experiments aimed to isolate beneficial bacteria from the rumen of ruminants. Briefly, 0.5 g of freeze-dried GRET (Wedid Co., Ltd., Seongnam, Republic of Korea) was suspended in 5 mL of MRS broth (Catalog Number: 69966-500G) purchased from Sigma Chemical Co. (St. Louis, MO, USA), and 50 μL of the supernatant was quickly applied to a sealing tape-type medium for *Lactobacillus* culture, then covered to minimize air exposure. The medium was placed in a sealed incubator with 5% CO_2_ and cultured at 37 °C for 48 h. From the cultured microorganisms, only strains identified as Lactobacillus were selectively chosen. A secondary culture was performed under the same conditions, excluding bacteria. At least 12 strains were isolated, and the 16S rDNA sequences were determined. Based on previous results, phylogenetic analysis and microbial identification were conducted according to the 16S rDNA sequencing results, as described in the previous studies [42,43]. All bacterial strains were precultured in MRS broth at 37 °C for 48 h. Then, they were cultured under CO_2_ at 37 °C for 72 h, followed by two rounds of sub-culturing.

We isolated and identified two candidate strains that could be utilized as biomass and biological materials from GRET. To secure intracellular metabolites of the two secured strains, heat treatment was performed as follows. Heat inactivation was performed by treating the cultures at 80 °C for 30 min. To verify the inactivation of microorganisms by heat treatment, we conducted follow-up experiments after confirming through smear tests that both Lc and Ls were killed. After heat treatment, the cultures were centrifuged at 13,500 rpm for 10 min to separate the supernatant (metabolites) from the bacterial cells. Both the bacterial cells and the culture supernatants were then lyophilized for further use. LGG was purchased as a standard strain from the Korea Culture and Technology Research Institute (KCTC). AW 264.7 macrophage cells were obtained from the Korean Cell Line Bank (KCLB), Korea. The cells were cultured in DMEM supplemented with 10% fetal bovine serum (Sigma Chemical, St. Louis, MO, USA) and 2% penicillin–streptomycin. Cells were maintained in a humidified incubator at 37 °C with less than 5% CO_2_ and subcultured before experimental use.

### 2.4. Antioxidant Activity

#### 2.4.1. Ferric Reducing Antioxidant Power (FRAP) Assay

The FRAP assay was performed to quantify the antioxidant activity of the samples based on their reducing power as described in the previous study [39]. Briefly, the FRAP reagent was prepared by dissolving 0.625 g of 2,4,6-tripyridyl-s-triazine (TPTZ) and 0.162 g of ferric chloride (FeCl_3_·6H_2_O) in 0.3 M sodium acetate buffer (pH 5.0). The mixture was stirred thoroughly until complete dissolution. The prepared solution was then filtered using a 0.2 μm membrane filter to remove any impurities. The assay was conducted as follows. 30 μL of sample, 70 μL of distilled water, and 900 μL of FRAP reagent were combined to achieve a total reaction volume of 1 mL. The mixture was incubated at 37 °C for 4 min. After incubation, the absorbance of the reaction mixture was measured at 595 nm. A blank was prepared under the same conditions, using an equal volume of distilled water in place of the sample. The antioxidant activity was calculated using the following formula:

FRAP activity (%) = (1 − B/A) × 100(1)

where A is the absorbance of the sample reaction mixture, and B is the absorbance of the blank.

#### 2.4.2. ABTS Radical Scavenging Activity

To evaluate the antioxidant activity of the metabolites, the ABTS [2,2′-azino-bis (3-ethylbenzothiazoline-6-sulfonic acid)] radical scavenging assay was performed with a slight change in method [39]. Briefly, 7 mM ABTS solution (0.038 g/10 mL distilled water) was mixed with 7.35 mM potassium persulfate solution (0.019 g/10 mL distilled water) in a 1:0.5 (*v*/*v*) ratio. The mixture was incubated in the dark at room temperature for 16 h to generate ABTS radical cations (ABTS^•+^). The resulting ABTS^•+^ solution was diluted with 100% ethanol to an absorbance of 0.70 ± 0.02 at 734 nm. For the assay, 50 μL of each sample was mixed with 500 μL of the diluted ABTS^•+^ solution at a 1:10 (*v*/*v*) ratio. The mixture was then incubated in the dark at room temperature for 6 min, after which the absorbance was measured at 734 nm. A standard curve was prepared using 0.01% (*w*/*v*) ascorbic acid under the same experimental conditions. The antioxidant activity of each sample was calculated as the percentage reduction in absorbance compared to the blank, according to the following equation:

ABTS radical activity (%) = (1 − A/B) × 100(2)

where A is the absorbance of the sample reacted with the ABTS solution at 734 nm, and B is the absorbance of the blank (ABTS solution with solvent only). This calculation expresses the percentage of ABTS radicals scavenged by the sample.

### 2.5. Cell Viability

Cell proliferation and cytotoxicity were evaluated using the murine macrophage-derived cell line RAW264.7 (Korean Cell Line Research Foundation, LCLB No 40071 RAW 264.7, Seoul, Republic of Korea). Cells were seeded into 96-well plates at a density of 5000 cells per well in 100 μL of medium and preincubated at 37 °C in a humidified incubator with 5% CO_2_ for 24 h. Following preincubation, various concentrations of the test samples (10 μL per well) were added, and the cells were incubated for an additional 24 h under the same conditions. Cell viability was assessed using the Cell Counting Kit-8 (CCK-8; Dojindo, Kumamoto, Japan), according to the manufacturer’s instructions. After treatment, 10 μL of CCK-8 solution was added to each well and incubated for 4 h at 37 °C with 5% CO_2_. The absorbance of each well was measured at 450 nm using a microplate reader. The relative viability of cells was calculated as a relative value to the negative control group, which was treated with only medium, and not a sample-treated control group.

### 2.6. Quantitative Analysis of NO Production

RAW264.7 cells were seeded in 24-well microplates at a density of 5 × 10^4^ cells/mL and incubated at 37 °C in a 5% CO_2_ incubator for 24 h. The cells were then treated with 100 μL of each sample along with 2 μg/mL LPS. Total nitric oxide (NO) production was quantified using a Nitrite/Nitrate Assay Kit (Biomax, Seoul, Republic of Korea), following the manufacturer’s instructions. Briefly, 2–50 μL of samples and standards were added to a 96-well microplate and adjusted to a final volume of 50 μL with 1× Assay Buffer. Subsequently, 25 μL each of 1× Nitrate Reductase and 1× co-factor was added to each well, and the plate was incubated for 1 h at room temperature in the dark. After the initial reaction, 10 μL of Enhancer was added to each well and incubated for an additional 10 min under the same conditions. Then, 50 μL each of Griess Reagent I and II was added sequentially to each well. The final reaction was carried out for 10 min at room temperature in the dark, and the absorbance was measured at 540 nm using a microplate reader (InfiniteM200 Pro Nano-quant, TECAN, Untersbergstrasse, Austria).

### 2.7. Quantitative Analysis of TNF-α and IL-6

RAW264.7 cells were seeded in 24-well microplates at a density of 5 × 10^4^ cells/mL and incubated for 24 h at 37 °C in a 5% CO_2_ incubator. The cells were then treated with 100 μL of each sample and 2 μg/mL LPS. The levels of pro-inflammatory cytokines IL-1β, IL-6, and TNF-α were quantified using ELISA kits (Thermo Fisher Scientific, Waltham, MA, USA), according to the manufacturer’s instructions.

### 2.8. Statistical Analysis

All data in this study were obtained from at least three independent experiments. The significant results of each experiment were expressed as the mean ± standard deviation. The significance of the *p*-value was considered to be less than 0.05. For statistical analysis of the results of many repeated experiments, the result values are obtained through the ‘two groups for variance’. After that, if the value of (P (F ≤ f) one-sided test χ^2^) is 0.5 or more, the *t*-test: two groups assuming equal variances is obtained. In addition, if the value of (P (F ≤ f) one-sided test χ^2^) is 0.5 or less, the *t*-test: two groups assuming unequal variances is obtained. In addition, when necessary, the significant values of all experiments obtained in this study were expressed only as the mean value.

## 3. Results

### 3.1. Isolation and Identification of Lactic Acid-Producing Bacteria from GRET

**Figure 1:**
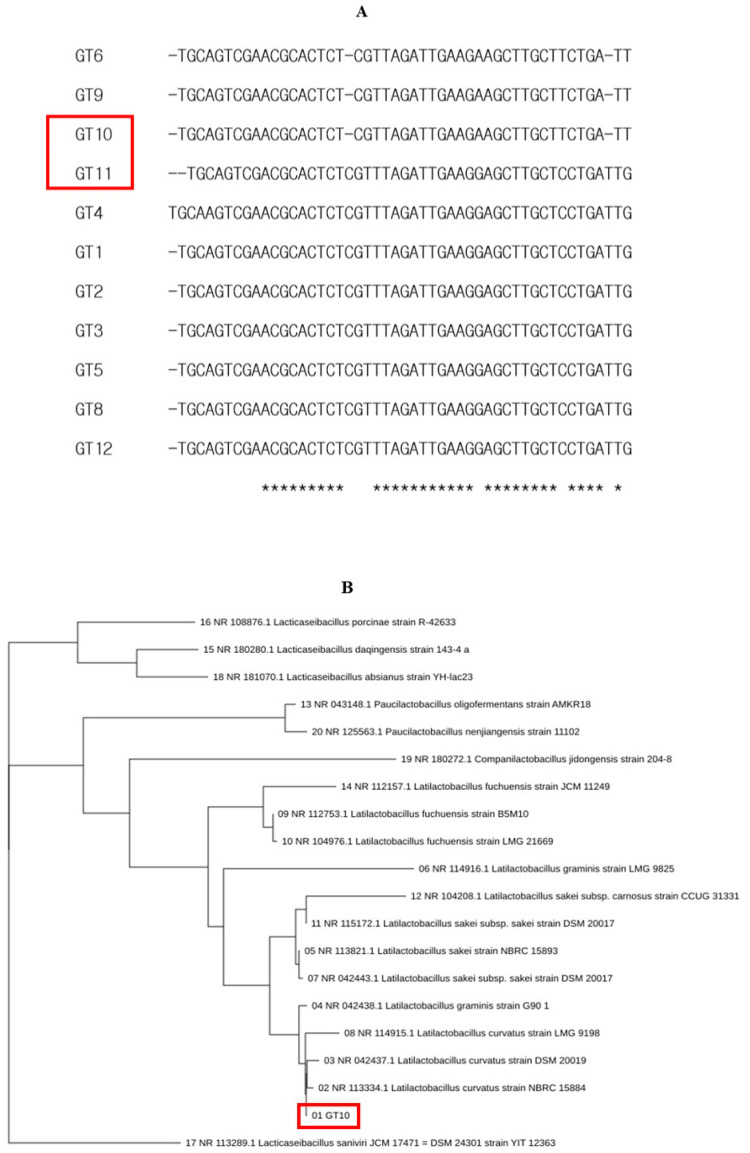

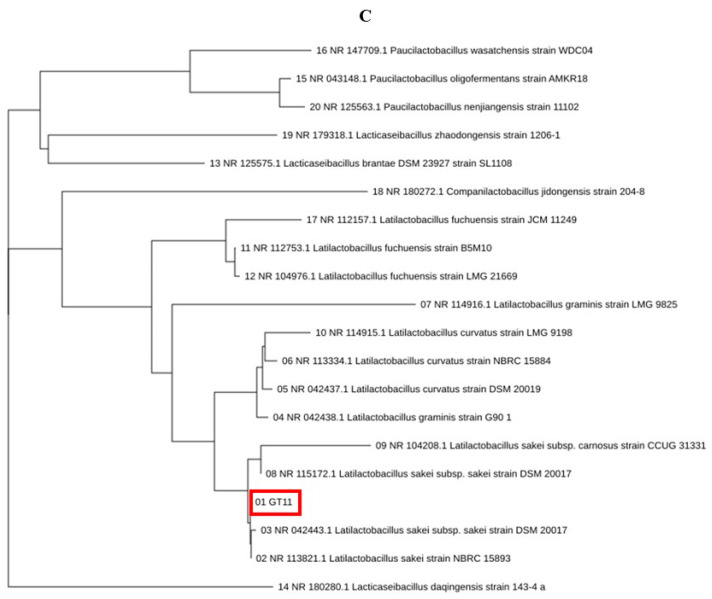
Phylogenetic analysis of lactic acid bacteria isolated from GRET based on 16S rDNA sequences. The 16S rDNA sequences of twelve strains (**A**) were determined and deposited (19 January 2025) in the Korea Biological Resource Center (Accession number: KCTC19209P) and a BLAST search (http://www.ncbi.nlm.nih.gov/BLAST/) was performed to compare the homology using CLUSTAL O (1.2.4) multiple sequence alignment software, as described in previous studies [41,42,43]. Phylogenetic analysis was conducted on all twelve strains, but considering the importance of lactic acid production, only GT10 (**B**) and GT11 (**C**) were selected for further study. The red box in the figure marks the microorganisms isolated in this study. “*” marks the bases that are important for homology comparison and indicates the conserved domain of the 16S rDNA of the corresponding microorganism.

The purpose of this study was to understand the types and functions of GRET, or intestinal beneficial bacteria. The main purpose of this study is to discuss the biological functions of unidentified microorganisms derived from GRET and their potential use as biomass. The isolated Lc and Ls showed the characteristic of lowering the pH of the culture medium from about 7.0 to 4.3 to 4.5 during culture among the 12 total isolated microorganisms. It is important to evaluate the biochemical characteristics for the production of other key probiotics in the isolation and identification of microorganisms. However, a faster and more powerful tool for microbial identification is the 16S rRNA (or DNA) base sequence and homology analysis. It is too time-consuming and laborious to evaluate all the key probiotic traits of countless microorganisms, so microorganisms were identified by performing only pH change measurement and 16S rRNA (or DNA) base sequence and homology analysis. Two strains, Lc and LS, were deposited in the Korea Biological Resource Center (Accession number: KCTC19209P). Using a simple method for isolating lactic acid bacteria, 12 single colonies were isolated from various microorganisms and re-cultured to isolate pure cultures. Among them, two strains were selected that had a fast culture speed and were not significantly affected by exposure to air. Through several repeated experiments, we isolated 12 different lactic acid bacteria from the first stomach of ruminants (GRET) from Korean cattle and further selected two beneficial bacteria. For the identification of two beneficial bacteria, 16S rRNA sequences were determined as described previously. Based on the 16S rRNA sequence analysis of 12 strains and their lactic acid productivity, GT10 and GT11 were selected and used in subsequent studies (Figure 1A). The microorganism named GT10 (Figure 1B) was 99% identical to *Latilactobacillus curvatus* (Lc) strain NBRS 15884, and the microorganism named GT11 (Figure 1C) was 99% identical to *L. sakei* (Ls) strain NBRC 15893, respectively.

### 3.2. Biochemical Characteristics of Bacterial Strains L. curvatus and L. sakei Isolated from GRET

**Figure 2:**
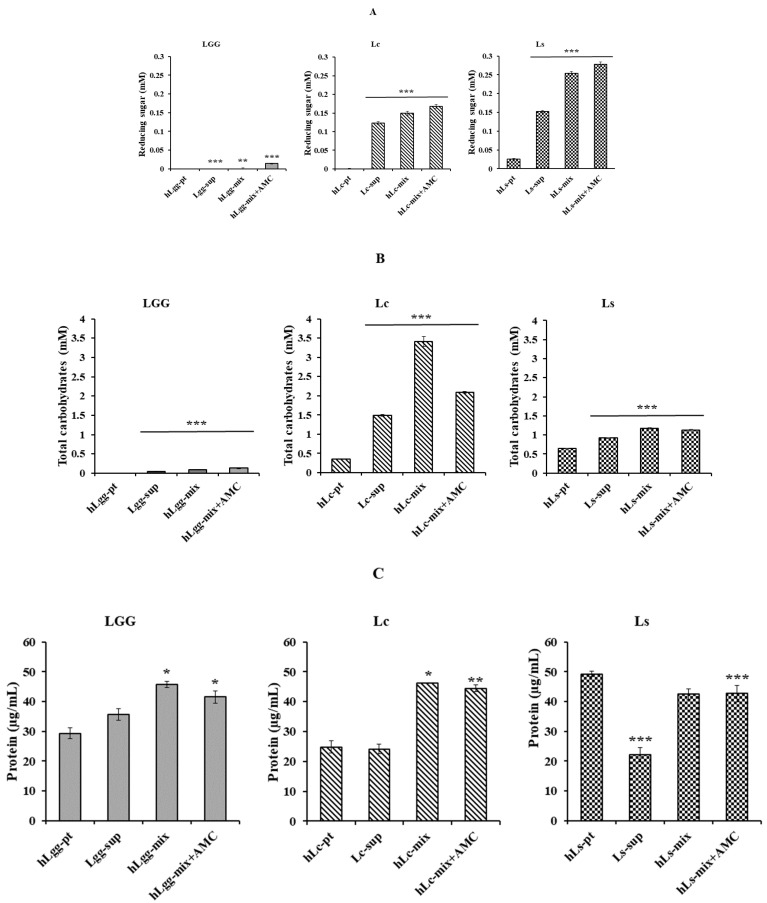
Biochemical characteristics of metabolites of GT10 and GT11. The reducing sugar (**A**), total carbohydrate (**B**), and protein content (**C**) of metabolites contained in the culture medium of each strain were evaluated. *L. rhamnosus* GG (LGG) is one of the most extensively studied probiotic strains and was used as a reference strain in this study. Based on the results of phylogenetic analysis, GT10 was named *L. curvatus* (Lc) and GT11 was named *L. sakei* (Ls), respectively. To expect their stability and synergistic effect, the cytotoxicity and bioactivity were verified by adding active molecular chitosan (AMC). Statistical probability compared to the control group is also indicated (***, *p* < 0.001; **, *p* < 0.05; *, *p* < 0.1).

To clarify the biochemical characteristics of the newly isolated strains in this study, an experiment was performed to confirm the composition of the metabolites of the strains. Total carbohydrate, reducing sugar, and protein quantification were performed according to previously described methods. The extracellular metabolites, according to the microbial culture of standard strains LGG, Lc, and Ls using selective media, were abbreviated as Lgg-sup, Lc-sup, and Ls-sup, respectively. After centrifugation (13,000 rpm, 5 min), each cultured cell was washed with PBS (pH 7.0) and suspended in MRS medium. After heat treatment, they were abbreviated as hLgg-pt, hLc-pt, and hLs-pt. Finally, the mixtures of extracellular metabolites and cells were abbreviated as hLgg-mix, hLc-mix, and hLs-mix, respectively. The results of the analysis of reducing sugar, total carbohydrate, and protein contents in each sample are presented (Figure 2A). As a result of the analysis of the supernatant components containing the metabolites excreted outside the cells, the total carbohydrate and reducing sugar contents were detected at higher levels than those of the control group, LGG. The concentration of reducing sugar in the Lc mixture (hLc-mix), i.e., including heat-treated dead cells, was measured to be 0.123 mM, total carbohydrate was 3.412 mM, and protein was 18.50 μg/mL. In contrast, the reduced sugar in Ls (hLs-mix), i.e., including heat-treated dead cells, was measured to be 0.254 mM, total carbohydrate was 1.175 mM, and protein was 19.84 μg/mL, respectively (Figure 2B). The reducing sugar and carbohydrate contents in both strains were detected to be higher than those in LGG, which was used as a control, which is thought to be due to the active metabolic activities of Lc and Ls. However, the protein content did not show a significant difference between the three strains and was evaluated at a similar level to LGG (Figure 2C).

AMC is an abbreviation for active molecular chitosan and was provided at the same concentration as the inner standard for quantitative analysis of total carbohydrate, reducing sugar, and protein contents. As shown in Figure 2, both the control and experimental groups added analytical significance to the sparse content of the control group in the quantitative analysis of reducing sugar and total carbohydrate, but it is thought to have a minimal effect in the experimental group as well. AMC is also known as chitosan oligosaccharide, and protein detection is not significant enough to be unclear, so it is thought not to affect the actual quantitative analysis (Figure 2A–C). The contents of extracellular metabolites of the two lactic acid bacteria isolated in this study showed a very significant difference compared to the control group. This is considered to have maintained or developed an activated metabolic function that has adapted for a long time under the rich carbohydrate supply conditions of GRET, unlike general lactic acid bacteria.

### 3.3. Effects of Lc and Ls on Cell Viability

**Figure 3:**
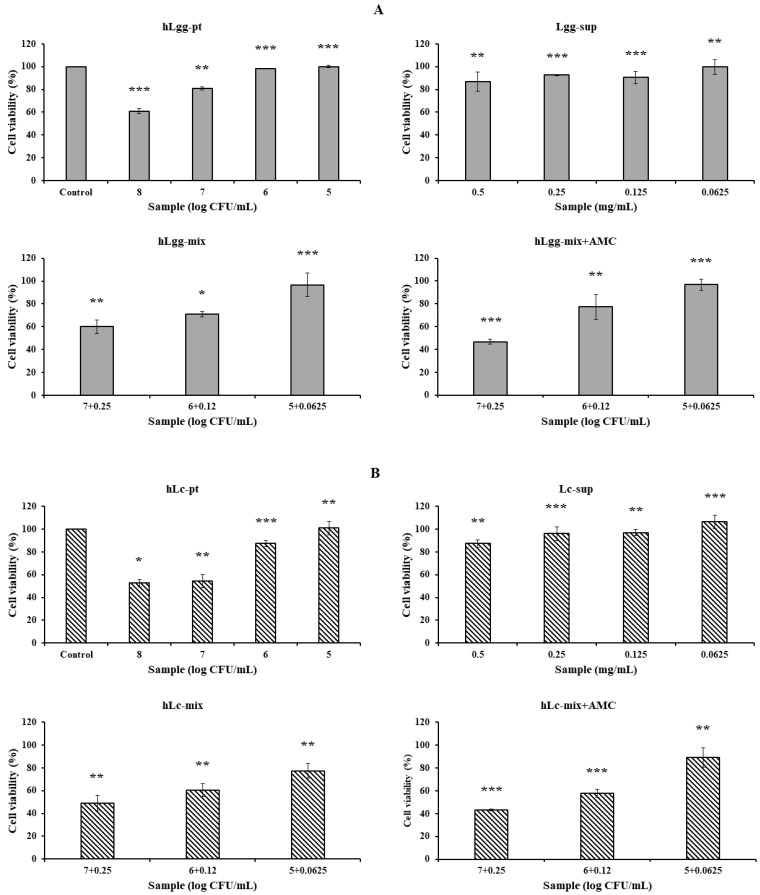

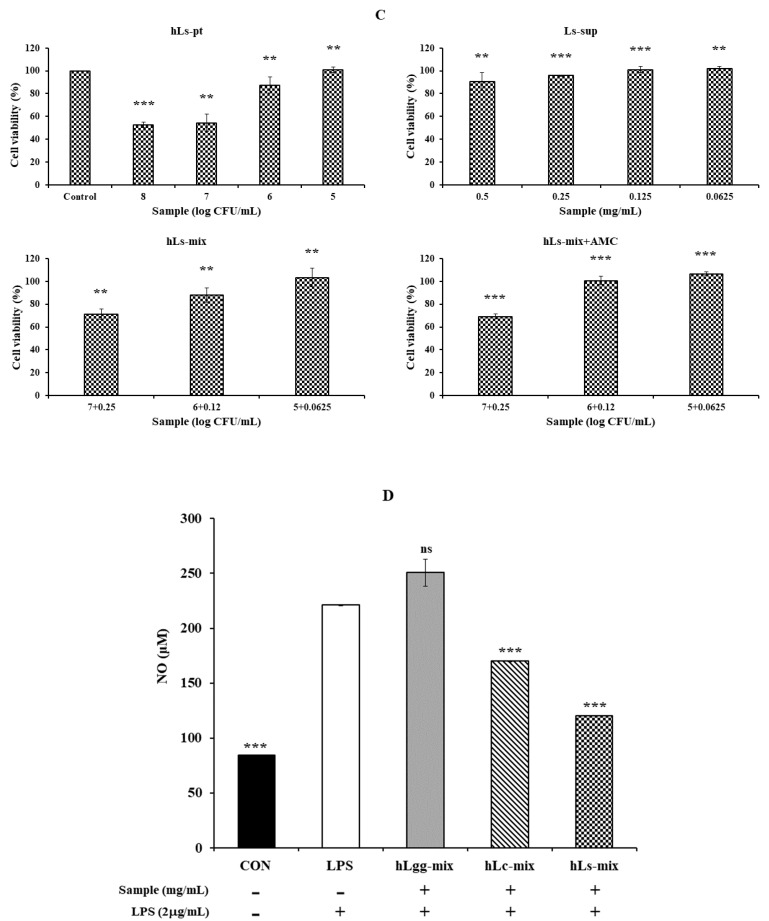
Cytotoxicity evaluation of cell metabolites and dead cells against macrophage RAW264.7. The cytotoxicity of the culture products of three types of lactic acid bacteria, LGG (**A**), Lc (**B**), and Ls (**C**), heat-treated dead cells, and their mixtures was evaluated to determine whether they can be used as prebiotics or probiotics. To evaluate anti-inflammatory activity, NO productivity was evaluated using LPS as a treatment control (**D**). Also, to expect their stability and synergistic effect, the cytotoxicity and bioactivity were verified by adding active molecular chitosan (AMC), as described in Figure 2. Statistical probability compared to the control group is also indicated (*p*-value, ***, 0.001; **, 0.05; *, 0.1).

The culture supernatant containing the control LGG and the metabolites of LC and Ls, and the precipitates obtained by centrifugation at 13,000 rpm for 5 min after heat treatment of each sample were designated as hLgg-pt, hLc-pt, and hLs-pt, respectively. The cytotoxicity of the culture supernatant (LGG, Lc, and Ls) and the heat-treated cell mixture was evaluated. The evaluation criterion was that the concentration at which cell viability was maintained at 90% or higher was considered non-cytotoxic. As a result, in the case of LGG, hLgg-pt was evaluated as not cytotoxic with a cell viability of over 90% at a concentration of 10^6^ CFU/mL. Lgg-sup also did not induce cytotoxicity at concentrations below 0.25 mg/mL. In these combined treatment groups (hLgg-pt + Lgg-sup), the cell viability was maintained at over 90% when treated with 10^5^ CFU/mL, indicating that no significant cytotoxicity was observed in either the single treatment or the combined treatment. However, when treated with 10^8^ CFU/mL, it was confirmed that the cell viability decreased by approximately 40% (Figure 3A). Similar to this result, it can be interpreted that the degree of cytotoxicity was somewhat lower than that of the control group when each sample of Lc and Ls was treated at the same concentration (Figure 3B,C). Based on these results, we concluded that the cytotoxicity of metabolites or cells of the control groups LGG, Lc, and Ls was not significant. More specifically, similar effects were observed in Lc, showing a similar level of cell safety to the LGG-treated group. On the other hand, Ls showed the characteristic of maintaining cell viability up to a higher concentration range under complex treatment conditions. Therefore, it suggests that the stability of Ls metabolites or cells themselves is relatively high. This suggests that lactic acid bacteria-derived macromolecules or cells themselves can be safely applied to food, feed, and various biological applications. In particular, it shows that using metabolites and heat-treated cells themselves as a biomass mixture does not induce cytotoxicity but rather improves functionality. One interesting point is that very similar results were maintained regardless of whether AMC was added or not. AMC has been reported to have very significant biological activities, such as anti-inflammatory, antimicrobial, and antioxidant activities, and by adding it, inhibition of metabolite degeneration and food preservation effects can also be expected.

### 3.4. Effects of Lc and Ls on NO and Inflammatory Cytokine Expression

Nitric oxide (NO) is a molecule that is important for cell signaling and is known to play an important role in various biological processes such as blood pressure regulation, insulin secretion, and peristalsis. NO is a bioactive free radical and an important small diffusible molecule involved in various biological activities. NO is a cytotoxic agent and a cell-transmitting molecule, and there is considerable interest in the production of NO by activated macrophages. This is because it plays an important role in exhibiting cytotoxic and cytostatic effects. Since NO plays an important role in the inflammatory process, it can be a potential target for the development of therapeutics for inflammatory diseases. Therefore, we evaluated the production of NO and the expression of inflammatory cytokines to evaluate the anti-inflammatory activity of the lactic acid bacteria isolated in this study.

Inducible nitric oxide synthase (iNOS or NOS II) is expressed at high levels only after induction by cytokines or other inflammatory factors. Overproduction of NO is associated with various inflammatory diseases, making it commonly used to screen for active substances that can modulate this pathway. The production of NO induced by lipopolysaccharide (LPS) in RAW264.7 cells was quantitatively evaluated. After LPS (2 µg/mL) treatment, NO production significantly increased. However, the inhibitory effects of the control group LGG and the metabolites of two types of lactic acid bacteria, Lc and Ls, and the cells themselves on NO production were not significant in RAW264.7 macrophages induced by LPS (Figure 3D). NO is a signal processor independent of various physiological activities, and its dysregulation is related to life span. Therefore, biological materials that reduce NO production by using specific inhibitors of inducible NO synthase may be of great help in improving various diseases. However, Ls or Lc used in this study did not show significant inhibitory activity regardless of the presence of AMC. This means that the enzymatic activity of iNOS, which is responsible for intracellular NO production, significantly increased after LPS treatment, but the cultured products of LGG, Ls, and Lc, or the heat-inactivated cell bodies themselves, did not inhibit the enzymatic activity of iNOS induced by LPS.

**Figure 4:**
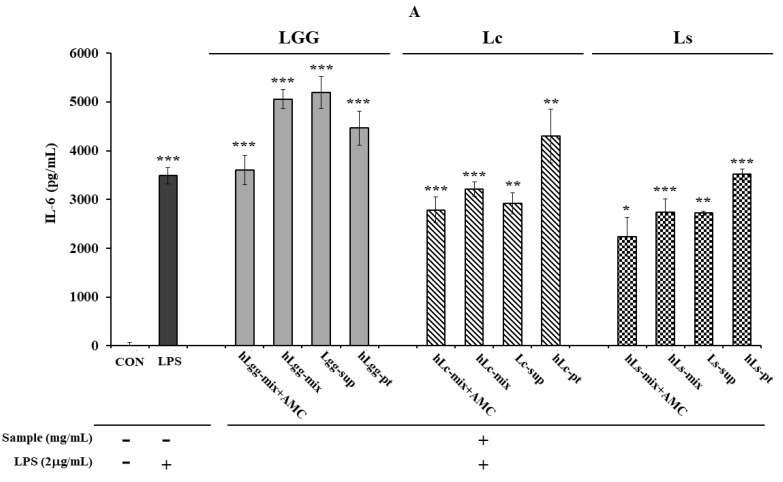

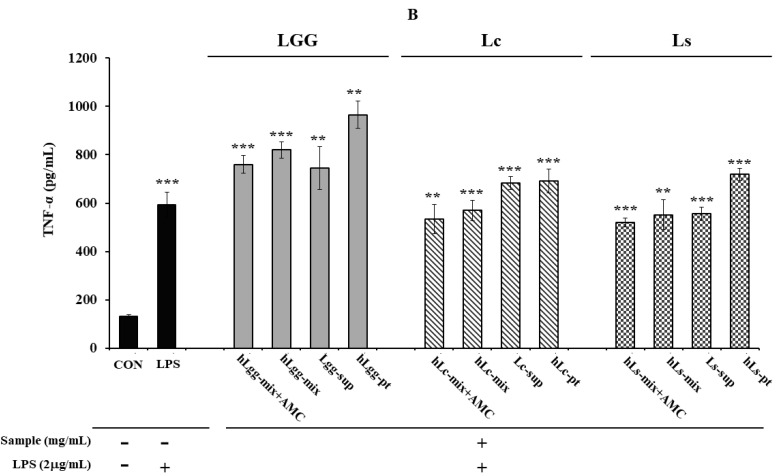
Evaluation of anti-inflammatory activity against the macrophage RAW264.7. The anti-inflammatory activity of metabolites, heat-treated dead cells, and the whole mixture of metabolites and dead cells of three types of lactic acid bacteria, LGG, Lc, and Ls, was evaluated by assessing the production of IL-6 (**A**) and TNF-α (**B)**, respectively. Statistical probability compared to the control group is also indicated (*p*-value, ***, 0.001; **, 0.05; *, 0.1).

On the other hand, after inducing an inflammatory response by treating RAW264.7 macrophages with LPS (2 μg/mL), the effects of heat-treated lactic acid bacteria or culture metabolites on the secretion of other major inflammatory cytokines, IL-6 (Figure 4A) and TNF-α (Figure 4B), were evaluated. When LGG was used as a control, and the metabolites, heat-treated cells, and mixtures of Lc and LS, which were lactic acid bacteria isolated in this study, were treated, it was confirmed that the amount of TNF-α secretion was significantly reduced compared to that following LGG treatment. Like the effect of reducing TNF-α secretion, a similar trend was observed in the reduction in IL-6 secretion or expression. The overall results showed that the metabolites derived from Lc and Ls, and the heat-treated cells, showed a significant inhibitory effect compared to the LGG-treated group. However, in the group treated with a mixture of metabolites and heat-treated dead cells, no significant reduction effect was observed compared to the LPS-treated control group. However, it was shown to be similar to or slightly lower than that of the LGG-treated group. These results probably suggest the need for precise concentration control to evaluate the concentration-dependent anti-inflammatory effect.

In addition, the AMC combination treatment group showed a more significant level of anti-inflammatory response inhibition effect than the single treatment group, suggesting the possibility of a synergistic effect between LGG and the metabolites of the other two lactic acid bacteria and heat-treated cells. Overall, both strains showed anti-inflammatory activity compared to LGG. In more detail, the Ls treatment group was evaluated to have a slightly higher inflammatory cytokine inhibitory effect than the Lc treatment group. When comparing the IL-6 expression levels, Ls showed an inhibitory effect about 2.61% higher than Lc in the AMC combined treatment group, and about 9.23% higher inhibitory effect was observed in the heat-treated killed cell treatment group. In the metabolite treatment group, Ls was evaluated to have a cytokine inhibitory effect about 18.47% higher than Lc (Figure 4A,B). As a result of comparing the level of TNF-α expression, Ls showed an inhibitory effect of about 25.0% higher than Lc. In addition, in the AMC combined treatment group, inhibitory effects that were 18.5% and 15.0% higher were observed, respectively, compared to the control LGG treatment group. On the other hand, in the metabolite treatment group, the difference between the two strains, Lc and LS, was relatively small, but Ls showed an inhibitory effect, about 6.8% higher than Lc. These results suggest that Ls exhibits overall better activity than LGG or Lc in suppressing inflammatory responses.

### 3.5. Antioxidant Effects of Lc and Ls

**Figure 5:**
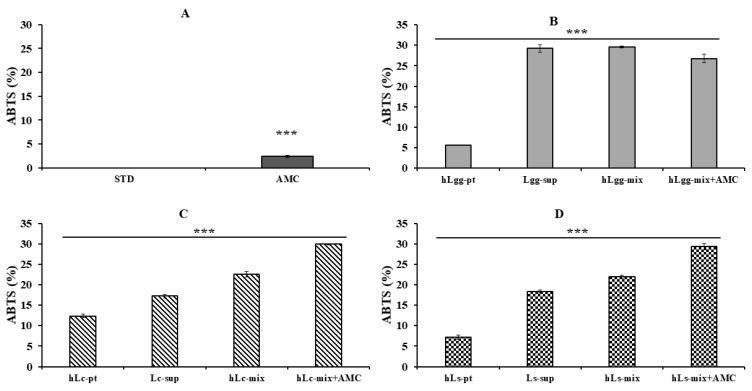
Evaluation of ABTS radical scavenging activity. The ABTS free radical scavenging activity of metabolites, heat-treated dead cells, and the whole mixture of metabolites and dead cells of three kinds of lactic acid bacteria, STD (**A**), LGG (**B**), Lc (**C**), and Ls (**D**), was evaluated. All data are expressed as the mean and standard deviation of more than three replicates. Statistical probability compared to the control group is also indicated (*p*-value, ***, 0.001).

The antioxidant activity of LGG, Lc, and Ls was compared and evaluated through ABTS radical scavenging activity analysis. The blank sample (STD), which was a negative control group, did not show any antioxidant activity, and the AMC (0.2 mg/mL) treatment group showed a low ABTS radical scavenging activity of approximately 5%, so it was used as a comparison group (Figure 5A). There was no significant difference in ABTS radical scavenging activity among the combined treatment groups of LGG-derived metabolites (Lgg-sup), heat-treated killed cells (hLgg-pt), and their mixture. There was a marked difference in antioxidant activity. However, the hLgg-pt alone treatment group showed a low activity of about 6%, and there was no significant difference under the combined treatment conditions of AMC (Figure 5B). In the case of Lc and Ls, the antioxidant activity significantly increased when treated with the complex group, including AMC, compared to the groups treated with metabolites and heat-treated cells alone (Figure 5C,D).

**Figure 6:**
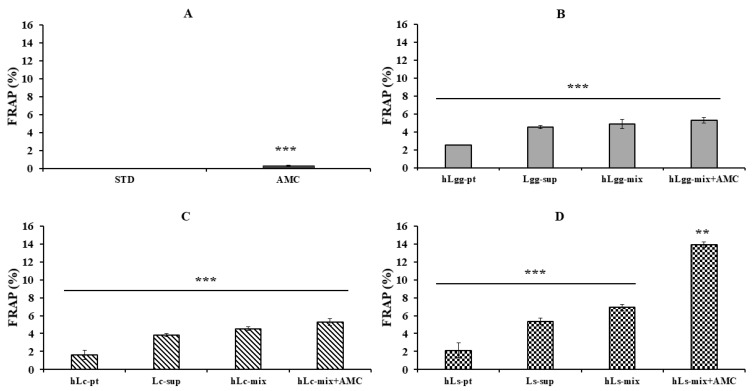
Measurement of antioxidant activity using FRAP. The metal-reducing antioxidant activities of metabolites, heat-treated dead cells, and the whole mixture of metabolites and dead cells of three kinds of lactic acid bacteria, STD (**A**), LGG (**B**), Lc (**C**), and Ls (**D**), were evaluated by FRAP. All data are expressed as the mean and standard deviation of more than three replicates. In addition, statistical probability compared to the control group is presented (*p*-value, ***, 0.001; **, 0.05).

Furthermore, the antioxidant activity of LGG, Lc, and Ls was evaluated based on the reducing power using FRAP analysis (Figure 6). It was performed under the same conditions as the ABTS radical scavenging activity test. Both LGG and Lc treatment groups showed similar FRAP activities depending on the selected sample conditions (Figure 6B,C). On the other hand, in the Ls treatment group, especially the combined treatment group with AMC, showed about 2–12% higher activity compared to the other samples (Figure 6D). These results suggest that Ls (Ls-sup and hLs-pt) have significant antioxidant activity against FRAP, and combined treatment with AMC can be expected to have a more excellent synergistic effect. It is thought that the synergistic effect is due to the chemical structural stability of AMC, its affinity with other compounds, and its binding stability. To supplement the additional significance of the addition of AMC, the concentration-dependent antioxidant, antibacterial, and excellent anti-inflammatory effects of AMC itself were verified [44,45,46]. Therefore, since the combined effect with AMC is greatly expected, it is thought that there is a high possibility that it will be used as a material for the development of functional foods or pet feed.

## 4. Discussion

In this study, we analyzed the antioxidant and anti-inflammatory activities of Lc and Ls, newly isolated lactic acid bacteria from GRET, to elucidate their biochemical properties. Probiotics are a group or species of microorganisms that provide beneficial effects to the human body. They are known to provide various health benefits through antioxidant and anti-inflammatory activities, as well as creating an environment that suppresses the survival of harmful bacteria in the intestine. In this study, both Lc and Ls were isolated from GRET-derived lactic acid bacteria, and their antioxidant and anti-inflammatory activities were confirmed through biological property analysis. These results suggest that these strains have potential as probiotics.

The RAW 264.7 cell line is the most commonly used cell line for screening anti-inflammatory and immunomodulatory compounds because it induces a strong inflammatory response to inflammatory stimuli such as lipopolysaccharide (LPS), a Toll-like receptor 4 (TLR4) agonist. TLR4 stimulation recruits nuclear transcription factors such as nuclear factor kappa B (NF-kB) and activates intracellular signaling cascades to produce inflammatory mediators or cytokines such as NO, TNF-α, and IL-6 [47,48,49]. CCK-8 and MTT assays showed that dead cells and metabolites from *L. curvatus* and *L. sakei* showed a cell viability of approximately 85% or more for RAW 264.7 cells, and when dead cells and metabolites were mixed, the cell viability was 95%. In another paper, the cytotoxicity of *L. sakei* isolated from kimchi heat-treated at 80 °C for 30 min against RAW264.7 cells was measured using the CCK-8 assay, and the cell viability was more than 90% in all treatment groups [29]. In addition, the cytotoxicity of *L. sakei* and *L. curvatus* isolated from kimchi heat-treated at 80 °C for 30 min against RAW264.7 cells was measured using the MTT assay, and the cell viability was more than 90% in all treatment groups [31]. In this study, we confirmed that the cytotoxicity results of two newly isolated lactic acid bacterial metabolites from GRET, heat-treated dead cells, their mixture, or their combination treatment with AMC were very similar to those previously published results. However, through this study, we present for the first time the importance and differentiation of antioxidant and anti-inflammatory activities in combination treatment with GRET-derived metabolites and AMC.

To evaluate the anti-inflammatory activity, RAW 264.7 cells were stimulated with LPS, and then treated with LGG, heat-treated Lc and Ls heat-killed cells and metabolites, either individually or in combination, to quantify the inflammatory cytokines produced in RAW 264.7 cells. Major inflammatory cytokines are well known to be IL-1β, IL-6, and TNF-α. Although our results were somewhat different, both Lc and Ls suppressed the expression of inflammatory cytokines, suggesting that these strains have anti-inflammatory activity. Hyun et al. [31] measured the concentration of inflammatory cytokines produced by heat-treating *L. curvatus* and *L. sakei* at 80 °C for 30 min, and then treating LPS-activated RAW264.7 cells. IL-1β, IL-6, and TNF-α were all significantly increased compared to the LPS alone treatment group. At the immunological level, it is known that transient increases in inflammatory cytokines have the accompanying effect of enhancing the initial immune stimulation effect, confirming the similarity with the results we presented [31]. It is also worth noting that after heat-treating *L. sakei* at 80 °C for 30 min, the mRNA expression of inflammatory cytokines IL-1β, IL-6, and TNF-α and the production of inflammatory cytokines were regulated in RAW264.7 activated with LPS [31]. As a result, when *L. sakei*-dead cells were treated with RAW 264.7, the mRNA expression of the inflammatory cytokine TNF-α was measured, and it was confirmed that the mRNA expression was decreased. In addition, the production of IL-1β and IL-6 was also confirmed to decrease. As a result, it can be evaluated that *L. sakei* inhibits the mRNA expression of inflammatory cytokines in RAW264.7 and thus the production of inflammatory cytokines. However, it should be emphasized that the research was planned and performed in terms of industrial usefulness and increasing expected effects rather than presenting all the results presented by previous researchers. The similarity of the results is also encouraging in that it allows us to anticipate the utility value of the two strains of lactic acid bacteria isolated from GRET.

Antioxidant activity is the ability to remove or neutralize reactive oxygen species (ROS), which are major factors that induce inflammatory responses. When antioxidants remove reactive oxygen species, they can suppress inflammatory responses. In addition, since oxidative stress can promote inflammation and activate signals that can induce chronic inflammation, anti-inflammatory antioxidants can neutralize reactive oxygen species to alleviate inflammatory responses. From this perspective, we verified the antioxidant activity of GRET-derived lactic acid bacteria metabolites and heat-treated dead cells (Figure 5 and Figure 6).

As described in the results section, antioxidant activity was confirmed to remove approximately 30% of ABTS radicals. Our results suggest that these lactic acid bacteria have significant antioxidant properties. As described in the paper by Heng Li [50], when the antioxidant activity of *L. sakei* isolated from garlic pickle was measured after heat treatment, DPPH was 17.01% and ABTS was 87.39%. In addition, the major ROS, superoxide anion and hydroxyl radical, were scavenged by 13.27% and 5.20%, respectively. In addition, in the paper by MÜRÜVVET DÜZ, the DPPH scavenging activity of *L. curvatus* isolated from lactic acid bacteria was 58.38 ± 0.60%, and the hydroxyl radical was scavenged by 78.86% ± 0.73%. In addition, the superoxide anion radical was scavenged from 21.63 ± 1.32% to 7.22 ± 0.04% [51]. Among the 12 lactic acid bacteria derived from GRET, the metabolites and heat-treated dead cells of Lc and Ls isolated did not show a significant difference in antioxidant activity compared to the results of previous researchers [50,51]. It is inevitable to acknowledge quantitative differences in activity depending on differences in culture or treatment conditions. The reason why antioxidant activity for DPPH was not measured in this study is that low-pH changes due to the growth of lactic acid bacteria may affect the measurement of DPPH activity. In any case, based on the evaluation of the antioxidant and anti-inflammatory efficacy of Lc and Ls isolated from GRET in this study, we suggest their utilization as various industrial materials.

## 5. Conclusions

The results of this study confirmed that the dead cells and metabolites of Lc and Ls isolated from GRET maintained stability against RAW 264.7 cells, inhibited the expression of inflammatory cytokines and NO after LPS treatment, and exhibited excellent antioxidant activity against ABTS free radicals and FRAP. This suggests that Lc and LS can alleviate inflammation and inhibit the occurrence of inflammatory responses by removing reactive oxygen species (ROS). In addition, a significant accompanying effect was confirmed when AMC was added and used. Critical aspects of all the presented results include the absence of verification of the molecular properties of metabolites or cell components, the expression mechanisms of antioxidant and anti-inflammatory cytokines, and the mechanism of interaction with AMC according to changes in molecular weight. However, it is very encouraging that Lc and LS isolated from GRET, which may be discarded as biological environmental waste, were isolated from GRET and their biological activities were evaluated. The significance of this study suggests that if additional research results are obtained, it could be utilized as a probiotic and prebiotic material suitable for various functional food materials or pet food.

## Data Availability

The original contributions presented in this study are included in the article. Further inquiries can be directed to the corresponding author.
